# Obesity in prenatal medicine: a game changer?

**DOI:** 10.1007/s00404-023-07251-x

**Published:** 2023-10-20

**Authors:** Natalia Carmen Prodan, Markus Schmidt, Markus Hoopmann, Harald Abele, Karl Oliver Kagan

**Affiliations:** 1 Department of Prenatal Diagnosis, University Clinic of Obstetrics and Gynaecology, Calwerstr. 7, Tuebingen, Germany; 2 Clinic for Obstetrics and Gynaecology. Sana Kliniken, Zu den Rehwiesen 9-11, Duisburg, Germany

**Keywords:** Overweight, Obesity, Reproduction, Miscarriage, Pregnancy, Pregnancy complication, Preeclampsia, Gestational diabetes mellitus, Ultrasound

## Abstract

Obesity is recognized by the World Health Organization (WHO) as a disease in its own right. Moreover, obesity is an increasingly concerning public health issue across the world and its prevalence is rising amongst women of reproductive age. The fertility of over-weight and obese women is reduced and they experience a higher rate of miscarriage. In pregnant women obesity not only increases the risk of antenatal complications, such as preeclampsia and gestational diabetes, but also fetal abnormalities, and consequently the overall feto-maternal mortality. Ultrasound is one of the most valuable methods to predict and evaluate pregnancy complications. However, in overweight and obese pregnant women, the ultrasound examination is met with several challenges, mainly due to an impaired acoustic window. Overall obesity in pregnancy poses special challenges and constraints to the antenatal care and increases the rate of pregnancy complications, as well as complications later in life for the mother and child.

## What does this study add to the clinical work

**Table:** 

What is already known?	
Obesity is an increasingly concerning public health issue across the world and its prevalence is rising amongst women of reproductive age and pregnant women.	
What is new?	
Obesity in pregnancy presents special challenges and limitations in prenatal care and increases the rate of pregnancy complications as well as complications later in life for both mother and child.	

## Introduction

Overweight and obesity (OWO) are defined by the World Health Organisation (WHO) as abnormal or excessive body fat storage that may impair health [1]. The measure by which these conditions are assessed is the body-mass-index (BMI). A BMI of over 25 kg/m2 is defined as overweight, a BMI of over 30 kg/m2 as obesity, and a BMI over 40kg/m2 is considered severe obesity, see Table 1.

**Table 1:** Classification of Weight According to BMI

BMI (kg/m2)	Classification
At or below 18.5	Underweight
18.5–24.9	Normal weight
25.0–29.9	Overweight
≥ 30.0 (including > 40.0)	Obesity
> 40.0	Severe obesity

**Fig. 1:**
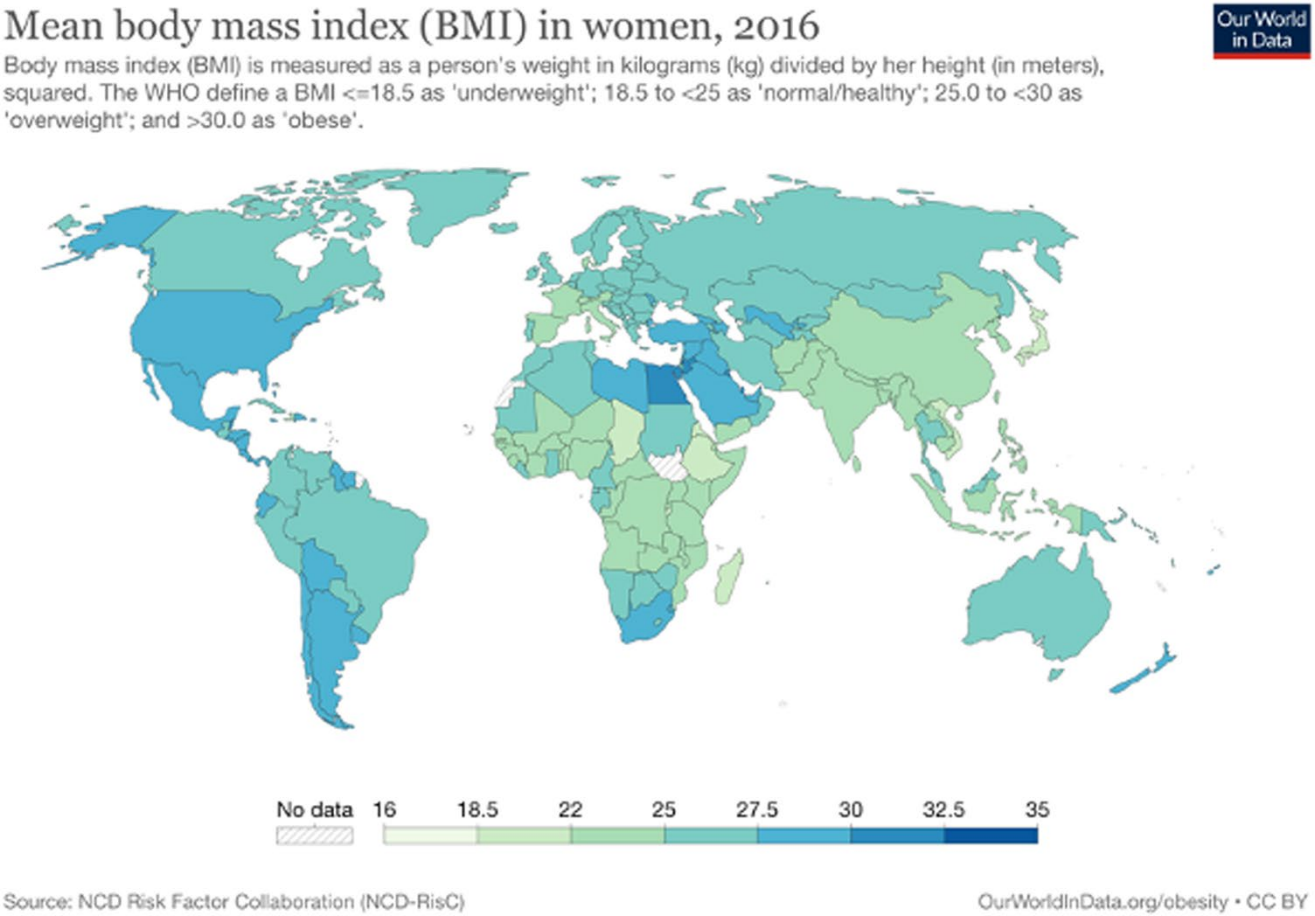
Mean BMI in Adult Women Across the World cf. [3]

According to WHO data, more than half of the world population is overweight or obese and the prevalence increases across the world every year, irrespective of gender and across most age-groups [2], see Fig. 1 [3]. In the European region the latest WHO report shows that over 50% of adults are afflicted by these conditions and that although women have a somewhat lower incidence of overweight at around 54% compared to men, they are more often afflicted by obesity (24% of women versus 22% of men) [1]. The latest statistics from Germany similarly confirms this worrying trend [4, 5]. Data from the United States of America (USA) indicate that between 1988 and 2018 there has been a marked overall increase in the proportion of OWO in the general population. In the female population specifically, this increased from a little over 51% in 1988 to about 69% in 2018 [6, 7]. Although the European and North-American regions have the highest prevalence of OWO, developing countries, such as India, are quickly catching up [8, 9]. This trend is also observed in other developing countries [10, 11].

## Epidemiology of overweight and obesity in women of reproductive age

Women of reproductive age (16–49 years of age) have seen an increase in the prevalence of OWO even before pregnancy. For instance, in the USA the prevalence of obesity in women of reproductive age has increased from 28.4% in 1999 to 2000 [12] to 41.5% in 2016 [13]. Similarly, in the United Kingdom (UK) it is estimated that 1 in 5 pregnant women are obese at the beginning of the pregnancy [14].

Obesity in women of reproductive age is becoming a serious public health issue, as this condition are associated with a decrease in fertility and an increase in pregnancy complications, as well as in long-term maternal effects and health issues in the offspring [15]. The last decades have also seen an increase in the transition of young women from normal weight to overweight and obesity [16].

## Health effects of overweight and obesity on mother and offspring

**Fig. 2:**
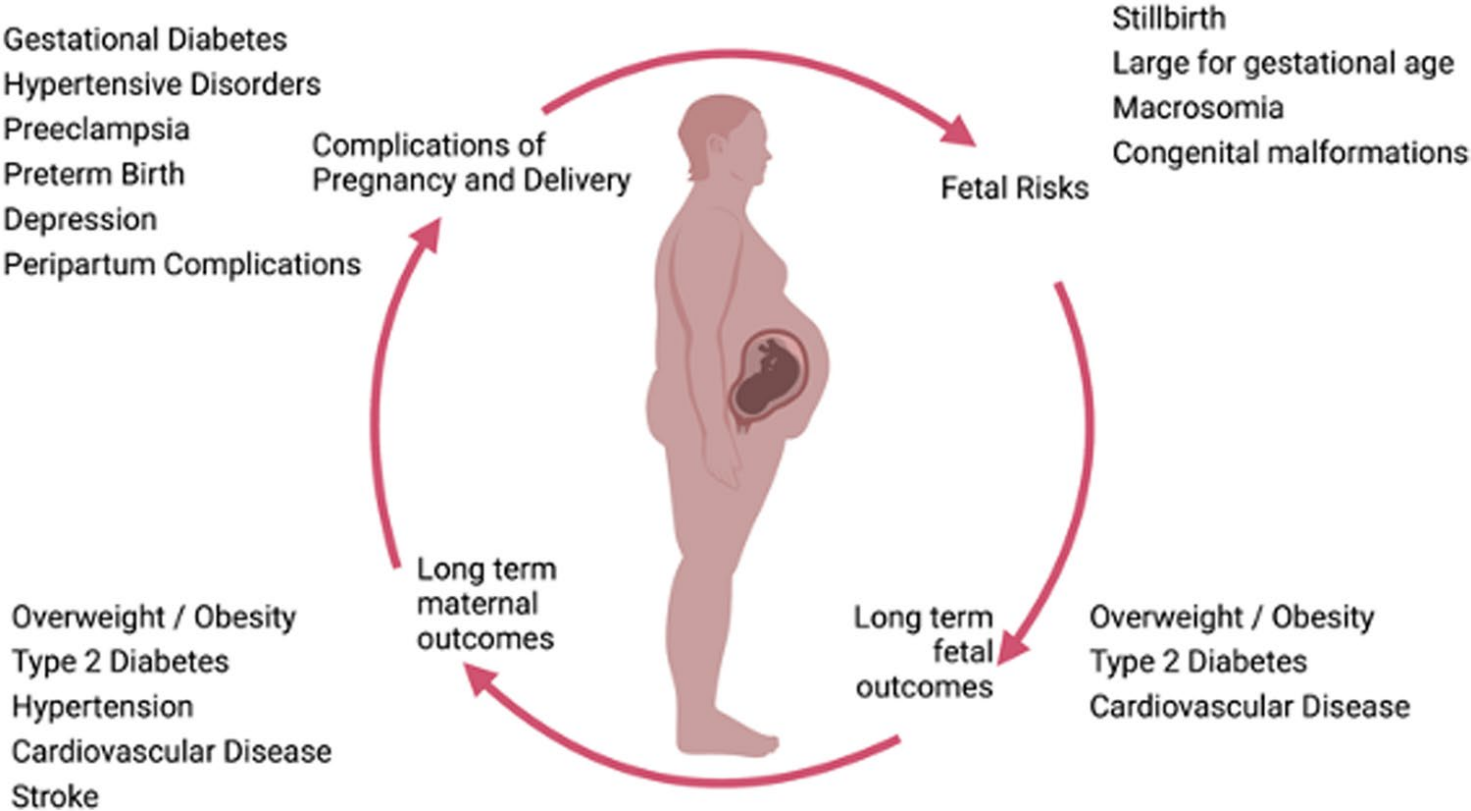
Effects of Obesity in Pregnancy and Later in Life (Created with BioRender.com. [23])

Obesity is associated with both short- and long-term health effects for women as well as for their offspring [17], see Fig. 2.

Existing research demonstrates that obesity impairs the whole reproductive cycle from fertility and conception to delivery [18]. Furthermore, it has been linked to the development of diabetes and cardiovascular disease later in life, both in the children born after in utero exposition to obesity [19, 20], as well as in the women themselves [21].

In utero exposure to an obesogenic environment leads to developmental programming which is likely to increase the risk of obesity in the offspring. Later on in life, additional environmental exposure of this offspring to factors which predispose to obesity, especially high caloric diet and lack of bodily activity, will only increase the likelihood of being overweight or obese as an adult [1].

In utero fetal exposure to obesity appears to lead to metabolic syndrome in the offspring, which suggests the presence of a chain reaction that transmits the metabolic disease from one generation to the next [22].

## Impact of obesity in reproductive medicine

The OWO-related factors that have an effect on the women’s and offspring’s health manifest themselves long before pregnancy. A woman’s fertility and capacity of maintaining a healthy pregnancy is influenced by her BMI.

Adipose tissue is a major endocrine organ and produces pro-inflammatory cytokines, as well as anti-inflammatory substances. Their balance can be severely affected in women with an increased amount of body fat. The insulin resistance and hyperinsulinemia, which are characteristic for obese women, cause a certain degree of hyperandrogenism. With the increased amount of adipose tissue, more androgens are aromatised to estrogens. Additionally, the sex-hormone-binding globulin (SHBG) and the insulin-like growth factor binding proteins are decreased. All these changes affect the balance between estrogens, androgens and SHBG and lead to a dysfunction of the hypothalamic-pituitary-ovarian axis [24]. Thus, these women experience ovulatory dysfunctions more often, leading to anovulatory cycles. There is also evidence that the disturbance in cytokine production in OWO women negatively affects the signalling pathways of the hypothalamic-pituitary-ovarian axis, as well as directly affecting the development of ovarian follicles and the maturation of oocytes [25].

The rate of spontaneous pregnancies is also greatly affected by BMI, decreasing by 4% with each BMI point above 29 kg/m2 [26]. Additionally, obese women are at higher risk of recurrent pregnancy loss, with some studies indicating a 75% increase in miscarriage compared to non-obese women [27].

The in-vitro fertilisation (IVF) procedures, which are otherwise a game-changer in women affected by low fertility, are proving challenging in obese women. Several studies demonstrated that the OWO patients have a lower percentage of live births when undergoing IVF cycles [28], and that this rate decreases as the BMI increases [29]. This decreased fertility rate is more pronounced in women with polycystic ovaries, but is independent of origin of the donor status (own or donated oocytes), which suggests a direct influence of the maternal organism. Even if the rate of implantation after embryo transfer appears to be similar in non-obese and obese women, their rate of miscarriage is higher, which leads to a lower live birth rate [30].

Two mechanisms have been proposed, which may responsible for this adverse outcome. On one hand the maternal metabolic alternations negatively influence the quality of the embryo, leading to a lower rate of blastocyst formation [31]. At the same time there is a lower implantation rate due to the modified endometrial gene expression, as well as endometrial transcriptome profile [32].

The complex mechanisms of successfully establishing and carrying a pregnancy to term appear to be disrupted in OWO women. Although the concrete underlying causes are not fully understood, the overall reduced pregnancy rate and higher miscarriage rate, both in natural conception and in IVF, in the context of the rising prevalence of obesity, is becoming a serious concern.

## Pregnancy complications in overweight and obese women

Adipose tissue is known to secrete a series of signalling substances, especially cytokines, including leptin, adiponectin, tumour necrosis factor-alpha (TNF-alpha) and interleukin-6 (IL-6). These influence the overall metabolism, as well as the function of several organs, especially the liver, the pancreas and the hypothalamic-pituitary-ovarian axis [33]. Pregnant OWO women, just like their non-pregnant counterparts, have a long-term positive energy intake leading to chronic adipocyte hypertrophy and adipogenesis. This in turn causes a low-grade chronic inflammation, which predisposes to insulin resistance.

Pregnancy normally induces adaptive mechanisms in the maternal body to allow for resource allocation to the fetus through carefully regulated adaptation, mediated by the placenta. Obesity changes this metabolic milieu by alterations in the glucose and lipid metabolism, as well as hormonal changes and inflammatory signalling. Additionally, the metabolic adjustments of pregnancy are due in part to the secretion of hormones belonging to the somatotropin family, like the human placental lactogen (hPL) and the placental growth hormone (hPGH) [34]. This adaptation is distorted in obese women, where the secretion of these hormones is affected [35–37].

Some essential metabolic adaptations of pregnancy are an increase in insulin resistance, an accelerated hepatic glucose production, as well as a surge in insulin release from pancreatic beta cells, to allow for an increased delivery of nutrients to the fetus [38]. Obese women have a significantly higher insulin level throughout the pregnancy. However, the increase in insulin resistance may lead to an elevation of glucose serum levels and abnormal glucose tolerance testing is more common.

Pregnancy modifies the lipid profile of the mother, causing a marked surge in triglycerides and total cholesterol and a moderate increase in low-density lipoproteins (LDL), especially in the third trimester, while the high-density lipoproteins (HDL) increase slightly from the first trimester onward [39]. In contrast to normal weight pregnant women, obese pregnant women have an atherogenic lipid profile with higher serum levels of triglycerides, total cholesterol, LDL and lower levels of HDL in early pregnancy. In the later stages of pregnancy however, the rate of increase of total cholesterol and LDL is less marked compared to pregnant women of normal weight, so that by the late second trimester normal weight women have higher cholesterol and similar LDL level compared to OWO women [40]. This slower rate of increase in lipids suggests a lack of adaptation to increased energy demands of the fetus in pregnancies affected by OWO.

Adipose tissue acts as an endocrine organ and thus OWO alter cytokine expression. Obese non-pregnant and pregnant women have high levels of pro-inflammatory cytokines such as TNF-alpha and IL-6 [41]. On the other hand, secretion of anti-inflammatory adipokines, the most important of which is adiponectin, is reduced. Adiponectin improves glucose metabolism and insulin sensitivity, as well as lipid metabolism [42]. The level of adiponectin normally decreases in pregnancy [43], but this decrease is more significant in obese pregnant women than in mothers of normal weight [44]. Leptin is another essential cytokine which acts in balance with adiponectin and regulates nutrients intake and energy expenditure [45]. Its level rises in a normal pregnancy and this increase is more pronounced in the obese pregnant women [46]. Thus, maternal obesity is associated with the placental disruption of the leptin-adiponectin interactions and the otherwise beneficial and necessary effects of these cytokines on placental development are reversed in obese mothers. Moreover, the level of expression of these cytokines is also altered in the fetus, which suggests an inter-generational pathway which causes an increase in the risk of obesity and insulin resistance in the next generation [45].

### Preeclampsia in overweight and obese pregnant women

Preeclampsia is one of the most significant complications of pregnancy. It has a multifactorial origin and is related to an inadequate trophoblast invasion and an abnormal maternal endothelial function, caused by very complex maladaptation processes, such as hypoxia, alterations of renin–angiotensin–aldosterone system, immunological maladjustment, oxidative stress. The result is the classical combination of maternal high blood pressure with end-organ dysfunction, which includes, along with many other short- and long-term problems, proteinuria, alterations of the renal and liver function test, placental insufficiency, and intrauterine growth restriction. These problems increase the risk of premature delivery, stillbirth, neonatal and long-term complications, as well as maternal acute and chronic health problems.

The mechanisms linking OWO and preeclampsia are not fully understood, but it is thought that the metabolic alterations induced by OWO play a significant role. One study conducted in rats has shown that hyperinsulinemia induces changes in the endothelial expression of nitric oxide synthase, which is implicated in the development of preeclampsia [47]. Oxidised LDL are markers of atherogenesis which results from lipid peroxidation of lipid and lipoprotein components of LDL [48]. They are found in obese women and decrease after weight-reduction [49]. It has been hypothesised that their concentration in the placenta is inversely correlated with the circulating levels of LDL [50]. The fact that the physiological increase in circulating LDL in second trimester is reduced in obese women might correlate with an increased level of oxidised LDL in the placenta. This may affect the development of extravillous cytotrophoblast, by inhibiting trophoblastic cell invasion, which is a marker of preeclampsia [51]. Moreover, both maternal obesity and preeclampsia show a strong correlation with and a similar pattern of increased cytokine expression, as shown in Table 2, adapted after [52].

**Table 2:** Common Metabolic and Inflammatory Features of Obesity and Preeclampsia

Feature	Obesity	Preeclampsia
Hyperinsulinemia	High	High
Insulin resistance	High	High
TNF-alpha	High	High
IL-6	High	High
Adiponectin	Low	Low
Leptin	High	High

The fact that maternal obesity is associated with an increased risk of developing gestational hypertension and preeclampsia is well known and was demonstrated in several studies and meta-analyses [53, 54].

O’Brien and colleagues analysed thirteen cohort studies, conducted in the USA, Sweden, the Netherlands, Latin and Caribbean America, Taiwan and the United Kingdom, including over a million pregnant women, and concluded that there is a linear correlation between BMI and the risk of developing preeclampsia. For an increase in BMI of 5–7 kg/m2, there is a twofold increase in the risk of preeclampsia in obese women [53].

Similarly, a Swedish population-based cohort study conducted between 1992 and 2006 confirmed this association [54]. The authors concluded that maternal obesity (excluding severe obesity) is correlated with an increase in the risk of early preeclampsia by 2.4, and over threefold for severe obesity.

Expansion of the definition of preeclampsia by the International Society for the Study of Hypertension in Pregnancy (ISSHP) in 2018 has led to an increase in the number of women diagnosed with preeclampsia; therefore, it is very likely that studies like the ones mentioned above would have shown an even stronger correlation between maternal obesity and preeclampsia [55].

The association of preeclampsia and maternal obesity is also observed in developing countries. A birth cohort study from China found a significant risk of preeclampsia in pregnant women with pre-pregnancy obesity (OR 3.78; 95% CI 2.65–5.41) [56]. Similarly, an eighteen-year observational cohort study from Reunion Island found a linear association of BMI and late-onset preeclampsia [57].

The Aspirin prophylaxis reduces the incidence of preeclampsia in high-risk women. Due to the increased risk of preeclampsia in OWO patients, the German guidelines recommend administering Aspirin starting in the first trimester in all patients with a BMI over 35 kg/m2 as a way of improving pregnancy outcomes [58].

### Gestational diabetes mellitus in overweight and obese pregnant women

Pregnancy induces a physiological insulin-resistance in the mother, which is meant to insure an adequate supply of nutrients to the growing fetus and placenta. The problem of the insulin-resistance in women with pre-pregnancy obesity is that their already overburdened pancreatic beta cells face an additional strain during pregnancy to increase insulin secretion to meet the demands of the fetus [34]. This imbalance has two major effects. It causes hyperinsulinemia and hyperglycaemia in the mother and leads to excessive exposure of the fetus to glucose, lipids and aminoacids. This results in fetal hyperinsulinemia, accelerated fetal growth and fat accumulation.

Obesity is thus a major risk factor for gestational diabetes mellitus (GDM), associated with fetal macrosomia, as several studies and meta-analyses have demonstrated [59–61].

Chu and colleagues conducted in 2007 an analysis of 20 studies from USA, Canada, Australia, Italy, France, United Arab Emirates, Israel, Finland, Nova Scotia and UK and showed that, compared to normal-weight women, the risk to develop GDM is two, four and eight times higher for overweight, obese and severely obese mothers, respectively [59].

A more recent meta-analysis focused on central obesity, assessed by abdominal subcutaneous fat thickness, waist circumference, waist-hip ratio, or body fat distribution. After evaluating 11 cohort studies totaling over 27,000 pregnant women, a strong correlation of this condition with GDM was found (OR 3.07, 95% CI 2.35–4.00) [60].

Alwash et al. looked for an association between GDM and obesity in general, as well as additional subtypes of central obesity and visceral adiposity. After evaluating 20 studies, which included approximately 50,000 women, they found an over 2.5 increase in risk for GDM for obesity [61]. Visceral adiposity showed an even stronger correlation of threefold risk increase.

**Fig. 3:**
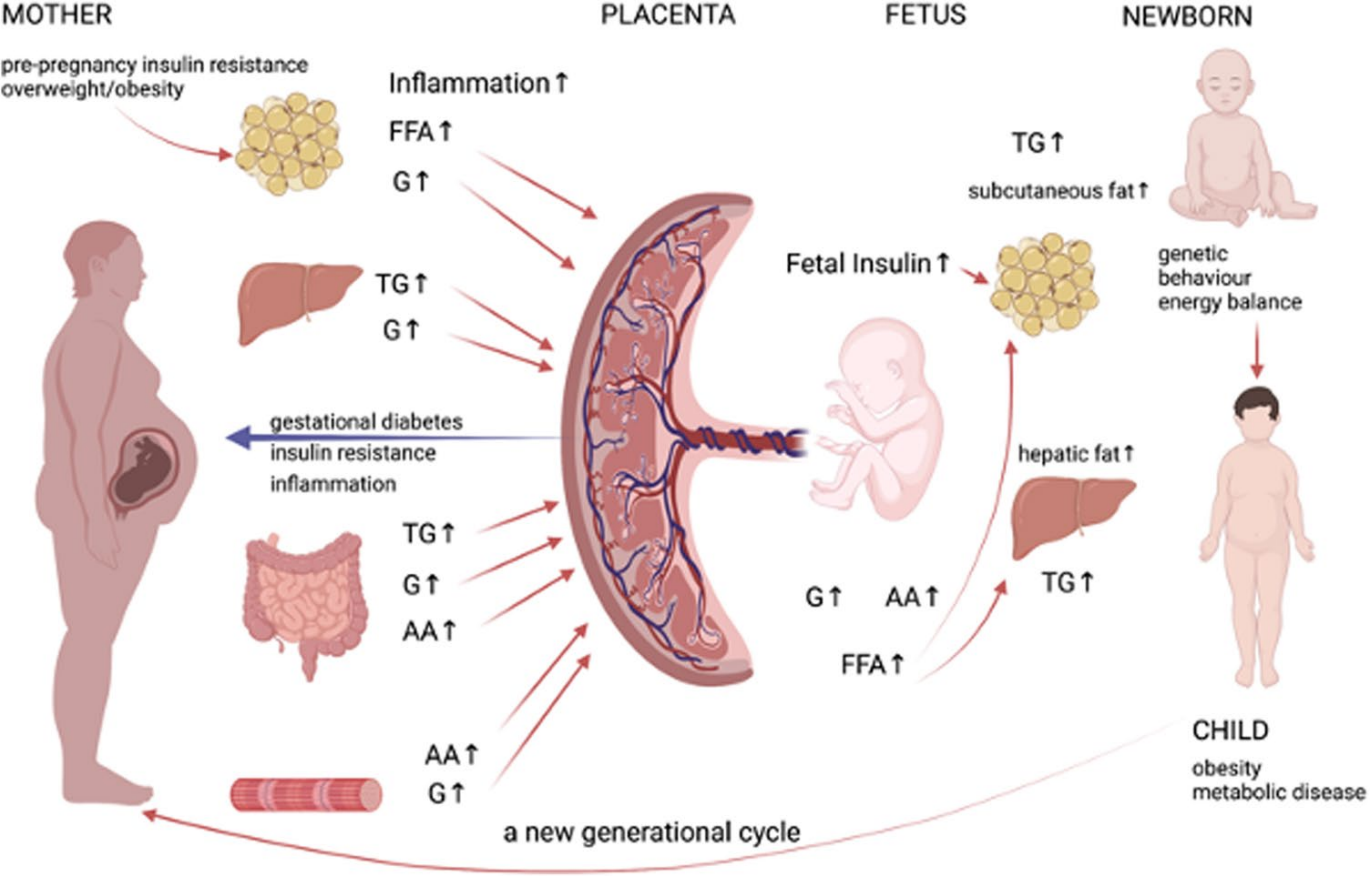
Effects of Obesity on Offspring, adapted after [34] (Created with BioRender.com. [23]). *FFA* free fatty acids, *G* glucose, *TG* triglycerides, *AA* aminoacids

Gestational diabetes mellitus has not only far-reaching consequences on the long-term health of the mother, by increasing her risk of diabetes mellitus, metabolic syndrome, and cardiovascular events later in life, but also on that of the offspring. This is thought to be caused by in-utero epigenetic and metabolic programming. The children born to mothers with obesity who develop GDM are at higher risk of being overweight and having insulin resistance themselves [17], see Fig. 3, adapted after [34]. Interestingly, maternal pre-pregnant BMI is a stronger predictor for childhood obesity than gestational diabetes [62]. Therefore, to minimise this intergenerational risk transfer, preconceptional counselling of women at risk is of outmost importance.

## Fetal abnormalities in overweight and obese pregnant women

Overweight and especially obesity pose significant challenges in the prenatal diagnosis of fetal abnormalities. They not only increase the risk of some fetal defects but at the same time, they make antenatal screening for chromosomal abnormalities and fetal malformations more difficult.

### Screening tests

The first trimester screening can be particularly difficult in OWO women, but the challenges posed by higher BMI regarding the sonographic evaluation of the fetal anatomy can be usually overcome by performing a transvaginal scan, as detailed in the next section. Moreover, the transvaginal approach might overcome some of the challenges encountered later on in pregnancy, during the second trimester anomaly scan, and as such, could be offered as an early anomaly scan [63].

Furthermore, the risk assessment for chromosomal abnormalities based on cell-free DNA non-invasive testing (NIPT) has a significant failure rate in OWO patients. This can be explained by an increased cellular turnover and apoptosis of adipocytes, as well as by the greater plasma volume of the OWO mother, leading to a dilution of the placental fraction of the cell-free DNA in maternal blood. The reported rates of no-call results vary greatly (5–70%) [64]; therefore, the true current no-call rate appears to be significantly higher than the approximately 1% rate commonly reported by most NIPT providers. Moreover, there is evidence that the cell-free DNA no-call rate increases by about 5% for each additional kilogram of maternal weight [65].

At the moment, there are no consensus recommendations from scientific societies across the world on this issue. However, some societies do recommend choosing other options for the screening for common trisomies other than NIPT in cases of severe maternal obesity [66].

### Increased prevalence of fetal malformations

The OWO pregnant women are at a higher risk for certain fetal structural abnormalities.

The altered pathways which may lead to a higher incidence of congenital abnormalities in fetuses of OWO mothers are not fully understood, but might be influenced by the higher levels of serum glucose often seen in OWO women periconceptionally and in the early stages of the pregnancy. Maternal hyperglycaemia leads to an increase of glucose levels in the embryonic environment, due to an overexpression of glucose transporters, as well as to oxidative stress and elevated free radical concentration, leading to a decreased expression of embryonic PAX3-gene and increased expression of the p53-gene [67]. As a consequence, apoptosis is increased which may be the link to the higher prevalence of fetal malformations. Moreover, alterations in the expression of critical enzymes in folate, homocysteine and glutathione metabolism influence the developing embryo heart. Reduced folate and glutathione and increased homo-cysteine levels are often seen in OWO women and could party explain the higher incidence of fetal abnormalities, especially congenital heart defects (CHD), in these cases [68].

Older epidemiological reviews have suggested an association of maternal obesity with various congenital malformations, such as spina bifida, heart defects, anal atresia, hypospadias, limb reduction defects, diaphragmatic hernia, and omphalocele [69–71]. More recent meta-analyses have confirmed this associations for some conditions, especially for CHD and neural tube defects (NTDs), as well as for orofacial clefts and hydrocephalus, but not for the others.

#### Congenital heart defects

The development of the fetal heart begins by the third week of gestation and by the eighth week, the cardiac structures of the embryo, as well as their functions, are mostly developed. The interaction between maternal metabolism and fetal development in this time frame is, therefore, most likely to explain the influence of maternal metabolism on the risk of CHD.

Several studies have looked for an association between an increased in CHD and either pre-pregnancy obesity or obesity during pregnancy. One large case–control study conducted in the USA between 1997 and 2007 found a 20% increase in the prevalence of CHD in women with pre-pregnancy obesity [72]. Interestingly, the strongest association was with hypoplastic left heart syndrome (OR 1.86, 95% CI 1.13–3.05). Strong associations were also found with left ventricular outflow tract defects (OR 1.27, 95% CI 1.02–1.59) and right ventricular outflow tract abnormalities (OR 1.43, 95% CI 1.20–1.69).

Cai et al. performed a meta-analysis of 14 studies investigating CHD and maternal obesity in 2013 and found a dose–response relation between the mother being overweight or having moderate or severe obesity and any fetal heart defects. They also confirmed the strong association of hypoplastic left heart syndrome and outflow tract defects [73] with maternal OWO. For severely obese pregnant women, the strongest observed association was for tetralogy of Fallot (OR 1.94, 95% CI 1.49–2.51). The correlation of CHD and maternal OWO was observed irrespective of maternal diabetes status.

Hedermann et al. performed a systematic review of 32 studies, which investigated the risk of CHD in women with various metabolic disorders associated with hyperglycaemia and insulin resistance, like maternal OWO, type 1 and 2 diabetes and gestational diabetes, hypertension, preeclampsia, dyslipidaemia and metabolic syndrome [74]. The authors noted the overall association between heart defects in the fetus and maternal obesity, but not in those that were overweight. However, they also pointed out that the evidence of associations with subtypes of CHD is contradictory.

Despite the heterogeneity noted in all aforementioned reviews, they all provide strong evidence, that indeed maternal obesity is associated with CHD in the offspring, and most likely some subtypes of CHD are more often observed than others.

#### Neural tube defects

NTDs stem from the impaired closure of the neural tube during the third and fourth weeks of pregnancy. The risk of NTDs in the fetus increases with increasing maternal pre-pregnancy weight, as well as with the obese status of the mother and this effect appears to be independent of folate supplementation [43].

Rasmunssen et al. have investigated the association of maternal obesity and NTDs in a meta-analysis which included 12 studies, most of them conducted in the USA. The authors concluded that obesity in pregnant women increases the risk for NTDs in the offspring 1.7 times, whereas severe obesity increased this risk over 3 times compared with mothers with normal weight [75].

A similar association was observed by Stothard et al. in their systematic review for all NTDs and obesity (OR 1.87, 95% CI 1.62–2.15). The authors noted that the effect was even greater for spina bifida, which was twice as high as the risk for other types of NTDs [71].

A recent meta-analysis of Vena et al. included ten studies published from 2000 to 2017 and noted a similarly strong association between obesity and NTDs in the fetus (OR 1.62 95% CI 1.32–1.99), but no correlation of these fetal malformations with overweight [76].

#### Orofacial clefts

Isolated orofacial clefts, including cleft palate and cleft lip and palate, are some of the more common congenital non-chromosomal abnormalities and require extensive surgical corrective treatment, as well as long term costly and time-consuming rehabilitation therapies. Given the negative impact this diagnosis can have on parents when discovered after birth, prenatal diagnosis and counselling of families is essential.

The association of OWO with orofacial clefts was noted by some authors [77], while others failed to note a correlation [78]. However, more recent pooled analyses did show an association. The study of Kutbi et al. found a correlation of BMI over 35 kg/m2 with a 36% increase in risk for orofacial clefts [79], while another group of authors found that not only obese, but also overweight pregnant women have a slightly increased risk of cleft lip and palate, but not cleft palate alone [80].

Due to the rising prevalence of OWO, as well as to the difficulty of diagnosing the orofacial clefts, these findings are of interest in prenatal diagnosis.

#### Malformations of the central nervous system

There are studies which show an association of congenital hydrocephalus with increasing maternal BMI. For instance, the meta-analysis of Stothard et al. reported a significant correlation of maternal obesity with hydrocephalus, with an odds ratio of OR 1.68 [71].

A recent study has shown that maternal obesity is associated with reduced cortical thickness in three frontal regions of the neonatal brain [81]. Although this imaging study was performed on a relatively low number of patients, the observation that maternal obesity might affect the neurological development of the offspring is concerning enough to warrant further investigation.

## Ultrasound for fetal abnormalities in overweight and obese pregnant women. Challenges and solutions

The detection of fetal congenital anomalies is achieved by prenatal ultrasound, an imaging method which can be challenging in cases of overweight and especially obese pregnant women [82].

**Fig. 4:**
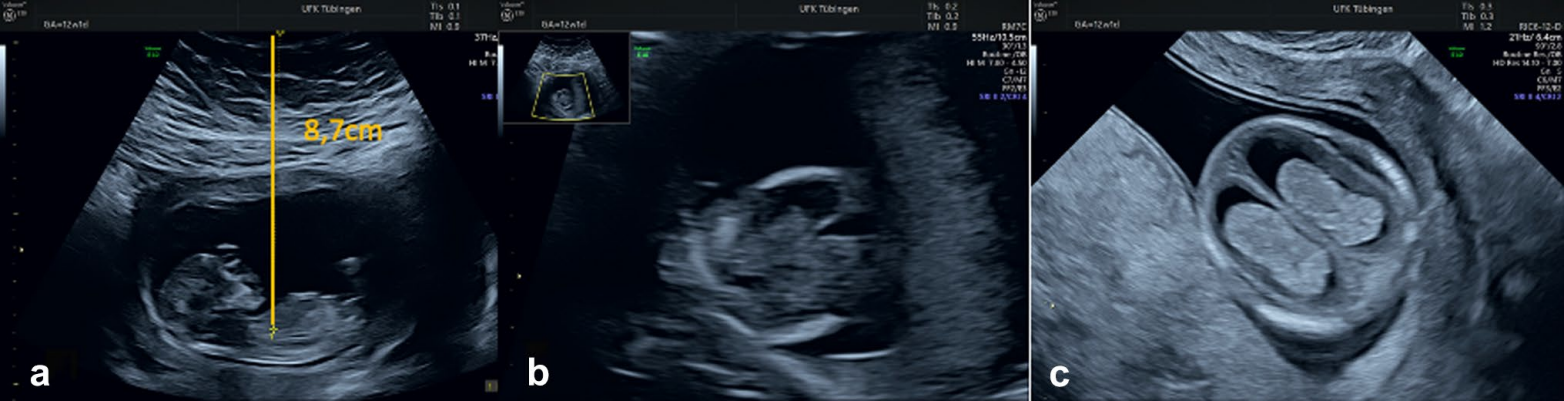
Ultrasound images of a fetus at 12 gestational weeks in a patient with a BMI of 38 kg/m2. Image **a** shows the crown-rump length and the distance between the fetus and the maternal skin of 8.7 cm. image **b** and **c** show the head in the same transverse section, obtained transabdominally (**b**) and transvaginally (**c**)

The cornerstone of the screening for congenital abnormalities is the second trimester scan, usually performed at 20–23 weeks of pregnancy. However, in OWO women, an early anomaly scan, performed transvaginally, could be also offered, as the transvaginal approach might improve visualisation of fetal structures, as seen in Fig. 4, and thus the detection of certain congenital malformations.

In obese women the excess of adipose tissue, especially around the abdominal area, will increase the insonation depth. This means that the ultrasound beam needs to travel longer to the region of interest and will suffer more absorption and dispersion in the surrounding tissue. When arriving at the region of interest, the ultrasound wave will have a lower energy and will have suffered more refraction, leading to more background noise. Consequently, the imaging quality is reduced, and some abnormalities may be missed even by the most experienced ultrasound diagnosticians.

To ascertain the level of suboptimal ultrasound assessment in obese women, Hendler et al. investigated a database of over 11000 pregnancies in the second trimester, beginning with 14 weeks of gestation, of which 38.6% were in obese women [83]. The authors concluded that obesity increased the rate of substandard visualisation of cardiac structures twofold, and by 31% for craniospinal structures. Repeat scans from 18 up until 22 gestational weeks improved the rate of visualisation of fetal structures, except for severely obese women, in whom the rate of impaired ultrasound assessment was about 50% at 18–20 weeks, with no improvement at later gestation.

A large trial including over 8500 singleton pregnancies, using the data from a prospective multicenter study in an unselected obstetric population (the FaSTER trial), also found that obesity lowers the likelihood of the diagnosis of fetal abnormalities. This was not due to fewer anomalies in that population, but due to a poorer performance of the ultrasound screening for congenital abnormalities in obese women [84].

Thus, from the perspective of prenatal ultrasound, it would be reasonable to consider the OWO or at least the obese pregnant woman as a high-risk patient and to refer her to specialised centres for a second trimester ultrasound assessment of fetal structures, including a fetal echocardiography. However, given the rising rate of OWO in the population of reproductive age women, this approach would generate enormous costs for health care systems.

There are approaches that may improve the quality of ultrasound image in obese women, and thus the rate of detection of fetal abnormalities.

Several technical adjustments are useful when scanning an OWO pregnant patient, as they can reduce to some extent the limitations of the impaired acoustic window. Lowering emission frequencies will improve penetration, but one has to bear in mind the output energy levels recommended for fetal scanning. The background noise can be reduced and the signal-to-noise ratio improved by employing tissue harmonic imaging, compound imaging and speckle reduction filters [82]. Tissue harmonic frequencies increase with depth, a feature which makes them useful in assessing fetal anatomy and in particular fetal heart [85]. Unfortunately, the harmonic frequencies will begin to decrease once an even greater depth is reached, due to tissue attenuation. Compound imaging uses the beam steering of the transducer array to rapidly acquire several frames from different angles, which will be integrated to form one real-time image, thus reducing acoustic artifacts and improving contrast resolution. Speckle noise reduction further improves image quality and contrast. Each of these techniques, alone or in combination can be used to improve the quality of ultrasound image. In fact, some newer ultrasound machines have dedicated setting specific to obese patients.

Another important aspect is how the diagnostician chooses to approach scanning of the obese patient and to plan the management in case of suboptimal visualisation. One option is to offer an initial transvaginal first trimester scan, which can assess the limbs, the abdominal wall, and the heart anatomy, followed by additional scans as needed until 20–22 weeks or even later to complete the fetal anatomical survey [86]. A second trimester transvaginal scan could improve the detection of certain fetal abnormalities, depending on the fetal lie [87]. Similarly, scanning through the anatomical acoustic windows offered by the umbilicus, the suprapubic area and both iliac regions, as well as scanning through a full bladder and repositioning the patient to optimize visualisation of fetal structures located the closest to these anatomical regions [82].

Of note is that offering repeat scans beyond the second trimester will not necessarily improve detection rate of fetal abnormalities, especially in severely obese women [83]. If an examination is not completed or fetal structures and organs cannot be adequately visualised, this must be recorded and communicated to the pregnant woman.

It is also important to note that the specialists performing the scans in obese women are at high risk of musculo-skeletal injuries and the rate of this problems is likely to increase in the future [82].

## Labour, delivery and postpartum complication in overweight and obese women

OWO pregnant women have a significant risk of serious peripartum complications, such as thromboembolism, organ failure, haemorrhage, infections, necessity for blood transfusions and mechanical ventilation, and even death. This was demonstrated in a recent retrospective cohort study from New York City [88]. Here, the rate of complication increased progressively with BMI and doubled for women with an BMI over 50 kg/m2 compared with normal controls.

### Preterm delivery

Preterm delivery is one of the major obstetrical complications and a leading cause of neonatal morbidity and mortality. In a Swedish cohort study the rate of spontaneous preterm delivery before 28 weeks, but not after 32 weeks, appeared to be increased in OWO women [89]. A recent meta-analysis has shown that the incidence of preterm prelabour rupture of membranes and extreme preterm birth (< 28 weeks’ gestation) increases with an increasing BMI [90]. Moreover, because the OWO pregnant women are at higher risk of pregnancy complication like preeclampsia and gestational diabetes, the rate of medically-indicated preterm birth is also increased. The Swedish cohort study concluded that the complication associated with obesity contribute to 60% of medically indicated deliveries before 31 weeks and 40% of those before 36 weeks of gestation [89].

### Fetal macrosomia and related complications

Macrosomic or large for gestational age (LGA) babies are a product of an excessive intrauterine growth. There is no standard definition of LGA fetuses. It is often considered to be a birthweight over 90th centile or over 95th centile adjusted for gestational age. A birthweight over 4000 g or, in some definitions over 4500 g is considered macrosomic. The cause of the excessive growth is likely the insulin resistance and the increased production of insulin-like growth factors in the obese mothers, which accelerate the uptake of nutrients in the placenta, as well as the up-regulation of transporters for glucose, amino acids and lipids, which make the nutrients more available to the fetus [38].

A systematic review of studies on maternal obesity and large fetal size found an increase of 142% of birthweights over the 90th centile in obese women [91]. The increase in neonates with a weight of over 4500 g at birth was even greater (277%).

As it is already well established in obstetrics, fetal macrosomia is associated with a wide range of fetal complications, such as shoulder dystocia, injury of the brachial plexus, fracture of the clavicle and/or humerus, hypoxia and hypoxic encephalopathy, hypoglycaemia. Furthermore, the risk of intrauterine fetal death near term is increased in obese women [92]. This concern leads to a higher rate of induction of labour (IOL). Additionally, a recent meta-analysis came to the overall conclusion that obesity is associated with the need for higher doses of prostaglandins and oxytocin and a higher rate of failed IOL [93].

The mother is at risk as well during delivery, with a higher rate of emergency caesarean section, instrumental delivery and failed instrumental delivery, obstetric haemorrhage, and injuries of the perineal tissue and anal sphincter. A cohort study conducted by Beta et al. showed that the composite maternal risk increases by about 4 percentage points at a birthweight above 4000 g and doubles for a birthweight of 4500 g or more, compared to controls. The risks for the neonates are even higher, with a tenfold increase in shoulder dystocia and fourfold increase in hypoxic encephalopathy compared to controls. [94]. Moreover, there is a 12-fold higher risk of neonatal complications for a birthweight above 4500 g compared to normal weight neonates.

### Caesarean delivery

As mentioned above, the rate of caesarean sections, especially emergency caesarean sections, is increased in OWO women. There are several factors that might contribute to this. These include a higher overall rate of complicated pregnancies in OWO women, as well as prior knowledge regarding the estimated fetal weight and the resulting concerns of obstetricians and midwives about shoulder dystocia, injuries and hypoxia.

A meta-analysis showed a 50% increase of caesarean section rate in overweight women and a doubling in obese women [95]. The induction of labour in OWO women also increases the chance of an unplanned caesarean section as compared to pregnant women with normal weight. This is especially true in nulliparous women, for whom there is an almost fourfold increase in risk [96].

## Pregnancy after bariatric surgery

It is presumed that bariatric surgery would improve the outcomes of pregnancy, by favourably changing the metabolic milieu in which the placenta and fetus develop. Indeed, the rates of preeclampsia, gestational diabetes and fetal macrosomia are reduced following bariatric surgery [97].

However, compared to matched-controls without prior surgery, these women still have a higher risk of congenital anomalies, preterm delivery and, unexpectedly, small-for-gestational-age (SGA) babies and perinatal mortality, as a meta-analysis from 2019 has shown [98]. The possible malabsorptive processes and nutritional imbalances after bariatric surgery might partly explain these findings. However, the authors noted that several confounding factors might explain the increase in risks after surgery. These include advanced maternal age and the fact that many women retain an unhealthy BMI even after surgery. In this population, there also appears to be an increased prevalence of unhealthy habits like cigarette smoking. Of note is that a recent nested case–control investigation based on the AURORA multicenter prospective cohort study in Belgium showed that targeted nutritional counselling and support for women after bariatric surgery reduced the rate of SGA neonates, thereby improving the outcomes of pregnancy in these women [99].

## Conclusions

OWO is an epidemic that is currently on the rise and places especially pregnant women at risk. This affliction is not only responsible for numerous serious complications in pregnancy but has far reaching consequences for the health of the mother and of the child. Through epigenetic changes it also affects the subsequent generations.

Fortunately, obesity is a condition that is amenable to interventions. Success largely depends on the close cooperation between the affected women and their healthcare professionals, as well as on the support these women receive, especially regarding pre-pregnancy weight loss.

OWO represents a challenge for prenatal diagnosis. There is a need for better algorithms to select pregnancies that are at an especially high risk. Appropriate interventions should be started before pregnancy and continued after delivery. We should search for methods that include metabolomics, proteomics, metagenomics, transcriptomics to identify novel biomarkers that will allow personalised approaches and targeted interventions to reduce complications not only during pregnancy but also later in life.
